# Sexual function and chemotherapy in postmenopausal women with breast cancer

**DOI:** 10.1186/1472-6874-12-28

**Published:** 2012-09-11

**Authors:** José Antônio Crespo Cavalheiro, AnaCristinadaCosta Bittelbrunn, Carlos Henrique Menke, Jorge Villanova Biazús, Nilton Leite Xavier, Rodrigo Cericatto, Fernando Schuh, Caroline Vieira Pinheiro, Eduardo Pandolfi Passos

**Affiliations:** 1 Breast Care Unit, Hospital de Clínicas de Porto Alegre (HCPA), Porto Alegre, Brasil; 2 Obstetrics and Gynecology Unit, Hospital de Clínicas de Porto Alegre (HCPA), Porto Alegre, Brazil; Faculdade de Medicina, Universidade Federal do Rio Grande do Sul, Porto Alegre, Brazil

**Keywords:** Sexual function, Breast cancer, Chemotherapy, Sexuality, FSFI

## Abstract

**Background:**

This cross-sectional, nested cohort study assessed Female Sexual Function Index (FSFI) scores in postmenopausal women with breast cancer receiving primary chemotherapy.

**Methods:**

The FSFI questionnaire was administered to 24 postmenopausal women one month after diagnosis of breast cancer (post-diagnosis group) and one month after completion of the first cycle of primary anthracyclin-based chemotherapy (post-chemotherapy group). Scores were compared to those of 24 healthy postmenopausal women seeking routine gynecological care (control group). All patients were sexually active at the time of enrollment. Mean age was 57.29 ± 11.82 years in the breast cancer group and 52.58 ± 7.19 years in the control group.

**Results:**

Scores in all domains of the FSFI instrument were significantly lower in the post-diagnosis group than in controls (−41.3%, p < 0.001). A further major reduction in FSFI scores was evident on completion of one cycle of primary chemotherapy (down 46.7% from post-diagnosis scores, p < 0.003), again in all domains. Six patients (25%) ceased all sexual relations, in a significant change from baseline (p < 0.001). After one chemotherapy cycle, a further five patients ceased sexual activity, for a total of 11 (45.8%) participants – a borderline significant difference (p = 0.063).

**Conclusion:**

The present study shows that female sexual function as assessed by the FSFI declines significantly at two distinct points in time: upon diagnosis of breast cancer and after administration of systemic chemotherapy.

## Background

Breast cancer is the second most common malignancy worldwide, and most frequently affects women over the age of 50 [1]. The disease is highly prevalent and incident in the Southern Region of Brazil. A total of 49,240 new cases of breast cancer are expected to have occurred in Brazil in 2010, for an estimated risk of 49.5 cases per 100,000 women. Breast cancer is the second most common malignancy in women (64.3 cases/100,000 population), exceeded only by non-melanoma skin cancers (87.4 cases/100,000), and is the most prevalent malignant neoplasm among women in the state of Rio Grande do Sul (81.6 cases/100,000). Fortunately, the number of new cases and the number of deaths are increasingly disproportionate as the population of breast cancer survivors experiences significant growth. This encouraging finding is largely due to early detection and to the therapeutic arsenal currently available [2,3].

Postmenopausal women experience reductions in estrogen levels and the classical symptoms of hot flashes, vaginal dryness, and dyspareunia, which contribute to a decline in sexual activity [4], also occurring on patients treated for breast cancer [5]. The detection of sexual dysfunction and poor sexual quality of life in women treated for breast cancer is the subject of increasing research interest. Unfortunately there is no clear relationship linking treatments applied to breast cancer patients and its direct effects on sexual dysfunction. Symptoms concerning to post-menopausal status or sexual function remains controversial on its origins, arising from intrinsic hormonal and/or psychological causes and can be worsened by chemotherapy for example [1,6,7].

Many patients with sexual complaints are seen by breast specialists and oncologists, all of which require specific attention. Patient’s quality of life and sexuality issues should be addressed in parallel with cancer treatment [3].

Female Sexual Function Index (FSFI) is a tool consisted by a brief scale designed for assessment of sexual functioning in women and was translated and validated for use in Portuguese-speaking patients in Brazil [7-9].

Primary chemotherapy, also known as neoadjuvant chemotherapy, is administered as first-line therapy before surgery. Its primary purpose is to reduce tumor size or extent in order to provide improved local conditions for surgery, often enabling conservative rather than radical procedures and, sometimes, even rendering inoperable tumors operable [10]. Initially restricted to patients with locally advanced or inoperable disease, primary chemotherapy has brought major advantages to the treatment of breast cancer. As it is administered before surgery or radiation, this model of treatment provides an opportunity for observation of in vivo response to chemotherapy.

The purpose of this paper is determine FSFI index at two specific moments for breast cancer patients – one month after the diagnosis and just before the second cycle of chemotherapy, comparing with FSFI index of postmenopausal patients on routine gynecological appointments. We believe that FSFI can be affected by chemotherapy and the diagnosis of breast cancer is enough by itself to cause a reduction on FSFI score.

## Methods

This cross-sectional, nested cohort study was conducted at the Hospital de Clínicas de Porto Alegre (HCPA) between July 2008 and March 2010, after approval by the local Research Ethics Committee. Nested cohort consist that patients with breast cancer were followed with FSFI index applied in two different moments as presented further.

The primary objective was to assess sexual function by FSFI in postmenopausal women after diagnosis of breast cancer and upon completion of one cycle of anthracycline-based primary chemotherapy (AC regimen, 60/600 mg/m^2^ IV every 21 days).

The study sample consisted of postmenopausal women who sought care at the outpatient Gynecology and Breast Care clinics of Hospital de Clínicas de Porto Alegre (HCPA), a public university hospital that cares for patients within the Brazilian Unified Health System framework. The case group comprised 24 postmenopausal women with recent diagnosis of breast cancer (treatment-naïve patients) with primary chemotherapy indication consecutively recruited from the Breast Unit service. The control group comprised 24 postmenopausal women with no known malignancy, recruited after presenting for routine gynecological care at the Obstetrics and Gynecology clinic that filled FSFI questionnaire. Patients were informed as to the purpose and objective of the investigation and provided written informed consent for participation and publication of the data obtained during the course of the study.

Inclusion was contingent upon the following criteria: postmenopausal status, absence of breast cancer, and absence of current hormone replacement therapy (control group) or diagnosis of breast cancer with indication for primary chemotherapy (case group); intellectual capacity sufficient to ensure understanding of the FSFI instrument; current sexual activity; and clinical performance status 0–2 considering ECOG levels [adapted from Zubrod et al. [11]].

The exclusion criteria to both groups were: presence of any other malignancies, prior diagnosis of psychiatric disorders, history of diabetes mellitus, vaginal surgery, hysterectomy or oophorectomy and sexual inactivity.

The Female Sexual Function Index (FSFI) questionnaire was administered to all patients. This instrument, which has previously been translated into and validated for Brazilian Portuguese, consists of 19 questions on the sexual activity performed in the last four weeks. It enables assessment of six sexual functioning domains: desire, arousal, lubrication, orgasm, satisfaction, and discomfort/pain [8,9]. Sub-domains are scored considering the values of each question and its respective conversion factors and total FSFI Score is the sum of the six results, ranging from 2 to 36 and better levels of sexual function are indicated by highest scores.

A demographic questionnaire on family, social, and educational history was also administered concomitant with FSFI to all patients in order to collect data on patient age, educational achievement (of the patient and her spouse or partner), number of marital partners, use of prior hormone replacement therapy, and family history of breast cancer.

In the case group, FSFI was administered 30 days after the appointment in which patients received the diagnosis of breast cancer and staging tests were performed during this period. After initiation of primary chemotherapy, just before the administration of the second cycle, patients were again submitted to FSFI questionnaire.

All patients invited to participate agreed to the project with no loss of follow-up.

Number of patients was determined considering a variation of 20% loss of FSFI score in the case group over control group with statistical power of 90% and the significance level was set at 5% (p ≤ 0.05).

Data were analyzed in the SPSS (Statistical Package for the Social Sciences) 17.0 software environment (SPSS Inc., Chicago, IL).

Continuous variables were expressed as mean ± standard deviation (in case of symmetric distribution) or median and interquartile range (for asymmetric distributions). Categorical variables were expressed as absolute and relative frequencies.

Student’s *t* test or the Mann–Whitney U was used for between-group comparisons of continuous variables. The Kruskal-Wallis test was used for comparison of educational achievement, tumor size, hormone receptor status, and histological grade.

Categorical variables were compared by means of Pearson’s chi-square test.

The Wilcoxon test was used for comparison of post-diagnosis and post-chemotherapy observations in the case group.

## Results

Mean age was 52.58 ± 7.19 years in the control group and 57.29 ± 11.82 years in the breast cancer group. Mean spouse or partner age was 52.54 ± 11.88 years and 55.96 ± 13.62 years respectively. Eighty-three percent of breast cancer patients had no family history of the disease. Sixteen patients (66.7%) were diagnosed with stage III and eight (33%) with stage II disease. Educational attainment did not differ significantly between either patient or partner group (Table 1).

**Table 1:** Sample characteristics

**Item**	**Controls**	**Post-diagnosis**	**p**
	**n (%)**	**n (%)**	
Age* (years)	52.58 ± 7.19	57.29 ± 11.82	0.102^†^
≤ 55	17 (70.8)	13 (54.2)	
> 55	7 (29.2)	11 (45.8)	
Educational attainment			
Primary education	18 (75)	17 (73.9)	0.839^‡^
Secondary education	4 (16.7)	3 (13.0)	
Higher education	2 (8.3)	3 (13.0)	
Partner age* (years)	52.54 ±11.88	55.96 ±13.62	0.359^†^
≤ 55	14 (58.3)	13 (54.2)	
> 55	10 (41.7)	10 (41.7)	
Partner education			
Primary education	15 (65.2)	15 (65.2)	1.000^‡^
Secondary education	6 (26.1)	6 (26.1)	
Higher education	2 (8.7)	2 (8.7)	
No. partners			
One partner	17 (70.8)	13 (54.2)	0.371^‡^
More than one partner	7 (29.2)	11 (45.8)	
Family history			
Negative	-	20 (83.3)	
Positive	-	4 (16.7)	
HRT			
No prior therapy	-	20 (83.3)	
Prior therapy	-	4 (16.7)	
Clinical stage			
IIA–B	-	8 (33.3)	
IIIA–B	-	16 (66.7)	
Tumor size			
T2	-	8 (33.3)	
T3	-	6 (25.0)	
T4	-	10 (41.7)	
Receptor / HER2/neu status			
ER+/PR+/HER2+	-	1 (1.4)	
ER+/PR–/ HER2+	-	0 (0)	
ER+/PR+/HER2–	-	9 (12.5)	
ER+/PR–/HER2–	-	4 (5.6)	
ER–/PR+/HER2+	-	0 (0)	
ER–/PR–/HER2+	-	2 (2.8)	
ER–/PR+/HER2–	-	0 (0)	
ER–/PR–/HER2–	-	8 (11.1)	
Histologic grade			
Grade I	-	1 (4.2)	
Grade II	-	17 (70.8)	
Grade III	-	6 (25.0)	

T2 = 2–5 cm; T3 = >5 cm; T4 = inflammatory tumor or invasion of chest wall or skin.

ER, estrogen receptor; HER2/neu, human epidermal growth factor receptor 2; HRT, hormone replacement therapy; PR, progesterone receptor.

*Mean ± SD.

^†^t-Student Test.

^‡^Pearson’s Chi-Square.

Mean FSFI scores were 23.0 in the control group and 13.5 in the post-diagnosis group. This significant difference (43% reduction, p < 0.001) was present across all test domains, and was most substantial in the desire, lubrication, and orgasm domains (Table 2).

**Table 2:** Comparison of FSFI scores in post-diagnosis patients and controls

**Domain**	**Controls**	**Post-diagnosis**	**Reduction**	p*
	**Median (IQR)**	**Median (IQR)**		
FSFI (overall)	23.0 (15.0 – 29.3)	13.5 (5.4 – 17.5)	41.3 %	<0.001
Desire	3.0 (2.4 – 3.6)	1.2 (1.2 – 4.0)	60.0 %	0.001
Arousal	3.5 (2.7 – 4.2)	1.8 (0.3 – 2.4)	48.6 %	<0.001
Lubrication	4.1 (2.5 – 5.4)	1.8 (0.3 – 3.2)	56.1 %	0.003
Orgasm	3.8 (1.3 – 5.1)	1.6 (0.3 – 2.4)	57.9 %	0.003
Satisfaction	4.2 (2.1 – 5.1)	2.2 (1.3 – 3.2)	47.6 %	0.001
Discomfort/Pain	11 (9.0 – 15.0)	8 (0.8 – 10)	27.3 %	0.006

*Mann–Whitney U. IQR, interquartile range.

After one cycle of chemotherapy, FSFI scores declined to 7.2 from 13.5 – a 46.7% reduction (p = 0.003). Pre and post-chemotherapy comparisons showed significant score declines across all domains, with particularly marked reductions in discomfort/pain and satisfaction domains (Table 3).

**Table 3:** Comparison of post-diagnosis and post-chemotherapy FSFI scores in breast cancer patients

**Domain**	**Post-diagnosis**	**Post-chemotherapy**	**Reduction**	p*
	**Median (IQR)**	**Median (IQR)**		
FSFI (overall)	13.5 (5.4 – 17.5)	7.2 (2.0 – 9.7)	46.7%	0.003
Desire	1.2 (1.2 – 4.0)	1.2 (1.2 – 1.2)	0.0%	0.001
Arousal	1.8 (0.3 – 2.4)	1.2 (0.0 – 1.2)	33.3%	<0.001
Lubrication	1.8 (0.3 – 3.2)	1.2 (0.0 – 1.2)	33.3%	0.001
Orgasm	1.6 (0.3 – 2.4)	1.2 (0.0 – 1.2)	25.0%	0.003
Satisfaction	2.2 (1.3 – 3.2)	1.2 (0.8 – 2.3)	45.5%	<0.001
Discomfort/Pain	8 (0.8 – 10)	1.2 (0.0 – 1.9)	85.0%	<0.001

*Wilcoxon test. IQR, interquartile range.

Table 4 shows FSFI score results stratified by demographic characteristics. Patients over the age of 55 scored worse than younger patients, particularly in the arousal, lubrication, and pain domains (p = 0.018, p = 0.030, and p = 0.047 respectively) (Table 5).

**Table 4:** Comparison of FSFI scores according to demographic characteristics

**Item**	**Controls**	**Post-diagnosis**	**Post-chemotherapy**
	**Median (IQR)**	**Median (IQR)**	**Median (IQR)**
Age (years)			
≤ 55	23.7 (15.9 – 29.1)**	15.0 (11.7 – 19.8)	8 (2 – 11.8)**
> 55	20.2 (9.5 – 29.6)*	8.0 (4.2 – 12.9)	2 (2 – 8)**
p^†^	0.383	**0.026**	0.063
Educational attainment			
Primary education	23.0 (15.4 – 28.4)***	8.8 (3.1 – 14.6)	7.2 (2.0 – 9.7)***
Secondary education	26.3 (6.8 – 32.8)	21.3 (20.7 – 25.0)	2 (2 – 15.9)
Higher education	21.6 (14.7 – 28.4)	15.0 (7.2 – 17.6)	7.2 (2 – 12.4)
p^‡^	0.805	**0.016**	0.889
Partner education			
Primary education	21.0 (14.3 – 28.0)**	12.8 (2.0 – 17.1)	2 (2 – 8)**
Secondary education	28.3 (17.2 – 32.1)	16.3 (13.2 – 21.3)	8.9 (5.9 – 13.3)*
Higher education	21.6 (14.7 – 28.4)	10.7 (7.2 – 14.1)	5.8 (2.0 – 9.6)
p^‡^	0.389	0.129	0.225
Partner age (years)			
≤ 55	27.0 (20.3 – 30.1)**	15 (11.7 – 19.8)	8 (2 – 11.8)**
> 55	18.7 (8.5 – 23.4)**	6 (2 – 12.8)	2 (2 – 8)*
p^†^	0.064	**0.001**	0.088
No. of partners			
One partner	23.7 (15.7 – 30.0)**	12.8 (5.7 – 17.4)	7.2 (2 – 8.8)**
More than one partner	21.0 (14.7 – 27.0)	14.6 (4.8 – 17.6)	7.2 (2 – 11.2)**
p^†^	0.349	0.608	0.820

*p ≤ 0.05, **p ≤ 0.01, ***p ≤0.001; vs. post-diagnosis group.

^†^Mann–Whitney U.

^‡^Kruskal-Wallis test.

**Table 5:** Comparison of FSFI domain scores according to patient age

**Domain**	**≤ 55 years**	**> 55 years**	p*
	**Median (IQR)**	**Median (IQR)**	
Desire	1.8 (1.2 – 2.7)	1.2 (1.2 – 1.8)	0.134
Arousal	2.4 (1.4 – 3.0)	1.2 (0.0 – 1.8)	0.018
Lubrication	3.0 (1.5 – 3.8)	1.2 (0.0 – 1.8)	0.030
Orgasm	2.0 (1.2 – 3.0)	1.2 (0.0 – 2.0)	0.150
Satisfaction	2.4 (1.6 – 3.2)	2.0 (1.2 – 2.8)	0.331
Discomfort/Pain	4.0 (2.6 – 4.8)	1.2 (0.0 – 3.6)	0.047

*Mann–Whitney U.

Women with lower educational attainment had worse FSFI scores on diagnosis. Significant differences were also found in the arousal, lubrication, satisfaction, and orgasm domains (p = 0.048, p = 0.012, p = 0.035, and p = 0.018 respectively), and were particularly marked upon comparison to the scores of women with a secondary-level education (Table 6).

**Table 6:** Comparison of FSFI domain scores according to patient educational attainment

**Domain**	**Primary**	**Secondary**	**Higher**	p*
	**Median (IQR)**	**Median (IQR)**	**Median (IQR)**	
Desire	1.2 (1.2 – 2.4)	1.8 (1.2 – 3.6)	2.4 (1.2 – 3.0)	0.454
Arousal	1.5 (0.0 – 2.3)^a^	3.3 (2.1 – 4.2)^b^	2.4 (1.2 – 3.0)^a,b^	0.048
Lubrication	1.2 (0.0 – 3.0)^a^	4.2 (4.2 – 4.8)^b^	3.0 (1.2 – 3.6)^a,b^	0.012
Orgasm	1.2 (0.0 – 2.0)^a^	4.0 (3.6 – 4.0)^b^	1.6 (1.2 – 2.0)^a,b^	0.018
Satisfaction	2.0 (1.0 – 2.6)^a^	4.4 (3.2 – 4.4)^b^	1.6 (1.2 – 3.2)^a,b^	0.035
Discomfort/Pain	2.4 (0.0 – 4.0)	4.8 (4.0 – 5.2)	2.8 (1.2 – 4.0)	0.134

*Kruskal-Wallis test.

^a,b^Same letters indicate no difference on Mann–Whitney U with an alpha of 0.05.

No significant differences were found in partner education. Women whose spouses or partners were older than 55 years, however, had significantly lower FSFI scores (Table 4).

Stratification of FSFI scores by tumor stage showed significantly lower scores in patients with more advanced disease (stages IIIA–B). The most significant differences were found in the desire and arousal domains (p = 0.004 and p = 0.038 respectively) (Table 7).

**Table 7:** Comparison of FSFI domain scores according to tumor stage

**Domain**	**IIA/B**	**IIIA/B**	p*
	**Median (IQR)**	**Median (IQR)**	
Desire	2.4 (2.0 – 3.0)	1.2 (1.2 – 1.7)	0.004
Arousal	2.4 (1.6 – 3.0)	1.4 (0.0 – 2.1)	0.038
Lubrication	3.0 (2.1 – 3.5)	1.2 (0.0 – 3.1)	0.061
Orgasm	1.8 (1.3 – 2.3)	1.2 (0.0 – 2.7)	0.350
Satisfaction	2.2 (1.6 – 3.2)	2.2 (0.9 – 3.1)	0.490
Discomfort/Pain	4.0 (3.0 – 4.6)	1.8 (0.0 – 3.9)	0.081

*Mann–Whitney U.

There were no statistically significant associations between FSFI scores considering prior diagnosis, histological grade, tumor size, or hormone receptor status (Table 8).

**Table 8:** Comparison of FSFI scores according to disease characteristics

**Item**	**Post-diagnosis**	**Post-chemotherapy**	**Reduction (%)**	p*
	**Median (IQR)**	**Median (IQR)**		
Clinical stage				
II A–B (n=8)	14.8 (14.5 – 19.0)	9.8 (7.2 – 12.1)	33.8	0.012
III A–B (n=16)	8.5 (2.6 – 16.4)	2 (2 – 8)	76.5	0.002
p^†^	0.052	**0.016**		
Tumor size				
T2 (n=8)	14.8 (14.5 – 19.0)	9.8 (7.2 – 12.1)	33.8	0.012
T3 (n=6)	10.5 (6.6 – 18.2)	4.6 (2.0 – 9.1)	56.2	0.028
T4 (n=10)	8.5 (2 – 15.6)	2 (2 – 8)	76.5	0.028
p^†^	0.301	0.132		
Receptor / HER2/neu status				
ER+/PR+/HER2+ (n=1)	8.8 (8.8 – 8.8)	8 (8 – 8)	9.1	-
ER+/PR–/ HER2+ (n=0)	-	-	-	-
ER+/PR+/HER2– (n=9)	12.8 (5.0 – 14.8)	7.2 (2 – 9.6)	43.8	0.018
ER+/PR–/HER2– (n=4)	18.5 (8 – 20.8)	6.0 (2 – 11.8)	67.6	0.068
ER–/PR+/HER2+ (n=0)	-	-	-	-
ER–/PR–/HER2+ (n=2)	11.4 (2 – 20.7)	2 (2 – 2)	82.5	0.317
ER–/PR+/HER2– (n=0)	-	-	-	-
ER–/PR–/HER2– (n=8)	13.5 (5.0 – 18.7)	5 (2 – 9.7)	63.0	0.018
p^‡^	0.688	0.677		
Histologic grade				
Grade I (n=1)	15 (15 – 15)	7.2 (7.2 – 7.2)	52.0	-
Grade II (n=17)	14.5 (7.6 – 18.5)	7.2 (2 – 10.6)	50.3	0.001
Grade III (n=6)	6.5 (2 – 15.8)	2 (2 – 8.4)	69.2	0.068
p^‡^	0.362	0.489		
Prior diagnosis				
No (n=19)	14.5 (4.2 – 17.6)	7.2 (2 – 9.9)	50.3	0.001
Yes (n=5)	8.8 (7.7 – 17.1)	2 (2 – 8)	77.3	0.043
p^†^	0.406	0.891		

T2 = 2–5 cm; T3 = >5 cm; T4 = inflammatory tumor or invasion of chest wall or skin.

ER, estrogen receptor; PR, progesterone receptor.

*Wilcoxon test.

^†^Mann–Whitney U.

^‡^Kruskal-Wallis test.

All study participants were sexually active at baseline. After diagnosis of breast cancer, 25% (n = 6) of case group had ceased sexual intercourse. This reduction was statistically significant (p < 0.001). After chemotherapy, a further five patients had ceased all sexual activity (n = 11, –45.8%); however, this difference was only borderline significant on comparison with the post-diagnosis group (p = 0,063).

Cessation of sexual activity was statistically associated with disease stage and tumor size in the post-chemotherapy group; patients with stage III A–B disease and/or T4 tumors were more affected than other patients in terms of new-onset sexual inactivity (Table 9).

**Table 9:** Association between tumor stage and size and sexual activity after diagnosis and after chemotherapy

**Variable**	**Sexual activity, post-diagnosis**			**Sexual activity, post-chemotherapy**		
	**Ceased (n=6) n (%)**	**Not ceased (n=18) n (%)**	p*	**Ceased (n=11) n (%)**	**Not ceased (n=13) n (%)**	p*
Tumor stage						
II A – B (n=8)	0 (0.0)	8 (44.4)	0.066	0 (0.0)	8 (61.5)	0.002
III A – B (n=16)	6 (100)	10 (56.6)		11 (100)	5 (38.5)	
Tumor size						
T2 (n=8)	1 (16.7)	7 (38.9)	0.352	1 (9.1)	7 (53.8)^†^	0.050
T3 (n=6)	1 (16.7)	5 (27.8)		3 (27.3)	3 (23.1)	
T4 (n=10)	4 (66.7)	6 (33.3)		7 (70.0)^†^	3 (23.1)	

*Pearson’s chi-square test or Fisher’s exact test.

^†^Significant adjusted residuals with alpha = 0.05.

## Discussion

This study assessed sexual function in postmenopausal women with breast cancer treated at a large, public university hospital in a developing country. FSFI scores were significantly reduced in women with breast cancer. In the immediate aftermath of diagnosis, patients already exhibited significant reductions in total score and across all FSFI domains as compared to controls. We can observe that merely receiving a diagnosis of breast malignancy is sufficient to produce a significant reduction on sexual function, even before patients have received any form of treatment. Survival-related concerns are highly influential at the pre-treatment stage and contribute to this decline in sexual functioning [12].

Administration of one cycle of primary anthracyclin-based chemotherapy was followed by another significant reduction in all domains of the FSFI score. The acute effects of chemotherapy—such as hair loss, weight gain, pallor, nausea, and vomiting—can make patients feel less attractive, and adverse effects such as vaginal dryness and itching contribute to reduced sexual function [13].

Patients over the age of 55 experienced more significant reductions in FSFI, as did women whose spouses or partners were older than 55. Maybe a greater distance from the borderline age of menopause can destabilize the couple’s sexual relationship, thus making it prone to the impact of cancer; however, further data are required to support this assertion. A diagnosis of cancer can also rekindle past conflicts between the couple, further contributing to a reduction in sexual function [14].

Although we found no significant association between reduction in FSFI scores and the prior diagnosis variable (treatment-naïve patients referred to our breast unit with clinical diagnosis of breast cancer), patients with advanced-stage disease experienced more significant losses in sexual function, particularly in the desire and arousal domains, which are more susceptible to the disturbances in self-image produced by locally advanced tumors.

Any discussion of female breast cancer must take into account not only the factors associated with cancer in general, but also the aspects related to the social function of the female body. In the field of symbolism, the breast plays fundamental roles in female identity, including sexuality and sensuality, as an object of pleasure and desire. A mutilated self-image disrupts the patient’s roles as spouse and mother and may give rise to feelings of helplessness, disgust and anguish [15-18].

Facing this crisis, which strikes at the physical, psychological, and social well-being of patients, requires adaptation to a new self-image. This, in turn, requires a supreme effort for which many women are unprepared. The challenges that arise upon diagnosis deeply unsettle the inner balance of patients and affect their relationships, making them more prone to conflicts at the personal and family level [16,19-21].

Breast cancer can affect three domains of female sexuality: sexual identity, sexual function, and sexual relations [16].

Although sexuality is often associated with sexual intercourse alone, it is far more than that: it is the combined experience of all changes that take place and affect the manner in which one views oneself and one’s body. Regardless of age, sexual function is dependent on one’s physical identity and psychic wellbeing, as well as on habitual sexual activity [3,20,22].

A diagnosis of cancer is a profoundly stressful event for patients and their families; all must adapt to the shock and uncertainty such a diagnosis brings. Patients, partners, and other family members may be affected by clinical-level depression and varying levels of anxiety, fear and other stress reactions [18].

Anticipation of changes in body image may be brought about by common-knowledge perceptions of the disease, including the possibility of breast loss or mutilation, hair loss, pain, and changes in reproductive capacity, as well as changes in perception of one’s health status, with fear of death and disease recurrence—compounded by fears as to the integrity of the intimate relationship and the possibility of partner rejection. All of these factors can have a negative impact on the sexual functioning of patients; even in the general population, depression and anxiety are associated with increased female sexual dysfunction [18].

Psychosocial support and assessment of quality of life must be integrated into the treatment and follow-up of women who receive a diagnosis of breast cancer. Chemotherapy has already been included as a quality of life variable in several studies, with some authors concluding it has no long-term impact on quality of life and others believing it is an important determinant of lower quality of life in survivors [15,17,23,24].

Time appears to be a determining factor of the influence of chemotherapy on quality of life after breast cancer treatment, its impact waning as years go by [1,7,12].

The adverse effects of breast cancer treatment, such as fatigue and nausea, must not be neglected. Patients who receive chemotherapy as part of their treatment regimens are apparently more likely to report severe, persistent fatigue than healthy women, affecting their sexual performance [22,25,26].

## Conclusions

The data presented herein attempt to isolate initiation of chemotherapy and diagnosis as milestones in sexual function. Future studies may seek a therapeutic approach capable of minimizing the negative effects of these events on sexual quality of life and on patients’ intimate partners.

There is still much to be observed and researched in terms of the partner as caregiver and of the partner’s ability to perceive losses in the woman [27].

Furthermore, health care providers are not properly trained to understand patient’s complaints or to obtain an adequate sexual history [28]. This is the key factor behind current difficulties in assessing sexual quality of life in cancer patients.

By selecting postmenopausal patients, we sought to exclude a number of variables that interfere with sexual functioning in premenopausal patients, such as hormonal changes and imminent loss of fertility due to cancer treatment.

FSFI is an adequate method for quantification of loss of sexual function in postmenopausal women, although the scale is unable to provide a satisfactory measure of the quality of the sexual relationship between subjects and their partners. Inclusion of only postmenopausal patients can consist in a limitation of this study, as well as a weak sexual/psychological analysis of those patients and their marital history. Our intention was observe and measure FSFI scores at diagnosis and after the first treatment (chemotherapy). We strongly believe that findings showed in this paper could stimulate more deep investigations and researches on breast cancer patient’s quality of life including their sexual problems.

## Competing interests

The authors declare no competing interest.

## Authors’ contributions

Conception and Design: JACC, EPP, Administrative Support: JACC, EPP, Collection and Assembly of data: JACC, ACdaCB, CHM, JVB, NLX, RC, FS, CVP, Data Analysis and Interpretation: JACC, ACdaCB, CHM, JVB, NLX, RC, FS, CVP, EPP, Manuscript writing: JACC, ACdaCB, CHM, JVB, NLX, RC, FS, CVP, EPP, Final manuscript approval: JACC, EPP. All authors read and approved the final manuscript.

## Pre-publication history

The pre-publication history for this paper can be accessed here:

http://www.biomedcentral.com/1472-6874/12/28/prepub
